# Examining the fundamental biology of a novel population of directly reprogrammed human neural precursor cells

**DOI:** 10.1186/s13287-019-1255-4

**Published:** 2019-06-13

**Authors:** Jan-Eric Ahlfors, Ashkan Azimi, Rouwayda El-Ayoubi, Alexander Velumian, Ilan Vonderwalde, Cecile Boscher, Oana Mihai, Sarathi Mani, Marina Samoilova, Mohamad Khazaei, Michael G. Fehlings, Cindi M Morshead

**Affiliations:** 1 New World Laboratories, Laval, Quebec, H7V 5B7 Canada; 2 Institute of Medical Sciences, University of Toronto, Toronto, Ontario M5S 1A8 Canada; 3 Division of Anatomy, Department of Surgery, University of Toronto, Ontario, M5S 1A8 Canada; 4 Division of Neurosurgery, Department of Surgery, University of Toronto, Ontario, M5T 1P5 Canada; 5 Institute of Biomaterials and Biomedical Engineering, University of Toronto, Toronto, Ontario M5S 3G9 Canada; 6 Division of Genetics and Development, Krembil Research Institute, University Health Network, Toronto, Ontario M5T 2S8 Canada; 7 Donnelly Centre for Cellular and Biomolecular Research, University of Toronto, Toronto, Ontario M5S3E1 Canada

**Keywords:** drNPC, Direct reprogramming, Neural precursor cells, Neural stem cells, In vivo neurogenesis, In vivo remyelination

## Abstract

**Background:**

Cell reprogramming is a promising avenue for cell-based therapies as it allows for the generation of multipotent, unipotent, or mature somatic cells without going through a pluripotent state. While the use of autologous cells is considered ideal, key challenges for their clinical translation include the ability to reproducibly generate sufficient quantities of cells within a therapeutically relevant time window.

**Methods:**

We performed transfection of three distinct human somatic starting populations of cells with a non-integrating synthetic plasmid expressing Musashi 1 (*MSI1*), Neurogenin 2 (*NGN2*), and Methyl-CpG-Binding Domain 2 (*MBD2*). The resulting directly reprogrammed neural precursor cells (drNPCs) were examined in vitro using RT-qPCR, karyotype analysis, immunohistochemistry, and FACS at early and late time post-transfection. Electrophysiology (patch clamp) was performed on drNPC-derived neurons to determine their capacity to generate action potentials. In vivo characterization was performed following transplantation of drNPCs into two animal models (Shiverer and SCID/Beige mice), and the numbers, location, and differentiation profile of the transplanted cells were examined using immunohistochemistry.

**Results:**

Human somatic cells can be directly reprogrammed within two weeks to neural precursor cells (drNPCs) by transient exposure to Msi1, Ngn2, and MBD2 using non-viral constructs. The drNPCs generate all three neural cell types (astrocytes, oligodendrocytes, and neurons) and can be passaged in vitro to generate large numbers of cells within four weeks. drNPCs can respond to in vivo differentiation and migration cues as demonstrated by their migration to the olfactory bulb and contribution to neurogenesis in vivo. Differentiation profiles of transplanted cells onto the corpus callosum of myelin-deficient mice reveal the production of oligodendrocytes and astrocytes.

**Conclusions:**

Human drNPCs can be efficiently and rapidly produced from donor somatic cells and possess all the important characteristics of native neural multipotent cells including differentiation into neurons, astrocytes, and oligodendrocytes, and in vivo neurogenesis and myelination.

**Electronic supplementary material:**

The online version of this article (10.1186/s13287-019-1255-4) contains supplementary material, which is available to authorized users.

## Background

There are currently two approaches for pre-clinical cell-based neural repair: (1) stimulation of endogenous neural precursor cell (NPC) reserves and (2) transplantation of exogenous cells. NPC transplantation is a promising therapeutic strategy to replace lost neural tissue, exert neuroprotective effects, and promote neuroplasticity [1, 2]. Human NPCs derived from fetal or embryonic tissue have been shown to promote recovery following rodent CNS damage [3–5]. However, these cell sources are burdened with ethical and safety concerns such as immune rejection [6, 7] and tumorigenicity [8]. To circumvent these issues, research has focused on induced pluripotent stem cells (iPSCs), which can be generated from one’s own somatic cells, as a source of NPCs [9–11]. Notably, the important role that iPSC technology plays in disease modeling, drug screening, and potential regenerative strategies cannot be disputed [12–17]. However, risks associated with iPSC technology such as genetic/epigenetic abnormalities and tumorigenicity continue to pose safety concerns [18–20]. Further, the process of generating NPCs from iPSCs is relatively cumbersome, requiring several weeks for the generation of iPSCs and directed differentiation to the NPC lineage [21, 22]. Thus, the manufacturing and cost challenges pose a challenge to the widespread clinical application of iPSC-derived NPCs in the treatment of neurodegenerative diseases.

To address these challenges, we have used a direct reprogramming method to produce NPCs from mature cell types without going through a pluripotent state [23]. Cells generated by direct reprogramming offer numerous therapeutic advantages over other cell sources. Similar to iPSC-derived NPCs, directly reprogrammed neural precursor cells (drNPCs) can be produced from cell sources that are accessible and outside the ethical concerns associated with cells generated from fetal and embryonic tissue. Additionally, drNPCs can be readily generated from autologous sources, thereby eliminating the possibility of graft rejection. Moreover, the elimination of a pluripotent mid-stage minimizes the risk of teratoma formation. The most common methodology for cellular reprogramming to a neural fate is viral vector integration, which has been used to generate various cell types within the neural lineage [24–28]. In spite of its wide adoption, viral vector integration carries the risk of genotoxicity associated with insertional mutagenesis [29, 30] which raises safety concerns that impede clinical translation. Furthermore, the reprogramming efficiency and purity achieved by these methods remain low [31].

Herein, we describe a novel population of drNPCs, generated from human somatic cells without the use of any integrating plasmids or viral vectors, and examine their in vitro and in vivo characteristics. Somatic cells were isolated and reprogrammed via transient expression of (i) Musashi-1 (MSI1), a neural RNA-binding protein that contributes to self-renewal of NSCs through the notch signaling pathway [32, 33]; (ii) Neurogenin-2 (NGN2), a helix-loop-helix transcription factor that has been previously used in different reprogramming paradigms to induce differentiation to neuronal fate [34–37]; and (iii) Methyl-CpG Binding Domain Protein 2 (MBD2), an epigenetic key-player that regulates neural differentiation and gene expression of cells through an HDAC-dependent mechanism [38, 39]. A vector expressing these three factors simultaneously was chosen based on preliminary screens we performed demonstrating the combinatorial effects of MSI/MBD2 as well as NGN2/MBD2 transfections. Herein, we show that mature human somatic cells, such as fibroblasts, keratinocytes, and bone marrow cells, can be directly reprogrammed to NPCs with the use of a synthetic plasmid that transiently expresses these three factors under an EF1-α promoter. This reprogramming method generates integration-free drNPCs with high efficiency within 2 weeks. We show that drNPCs are neurally committed and exhibit a neuronal electrophysiological profile in vitro. Following transplantation in vivo, drNPCs remain neurally committed and differentiate into neurons, astrocytes, and oligodendrocytes, while also migrating along the rostral migratory stream (RMS) to the olfactory bulb. This reprogramming paradigm provides a foundation to further study personalized medicine approaches and develop novel cell-based therapeutics using drNPCs. Moreover, this direct reprogramming approach can be adopted to generate any autologous neural cell type to treat CNS disorders.

## Methods

### Generation of drNPCs from bone marrow-derived cells

A human (male, 34 years old) bone marrow aspirate (25ml) with heparin anticoagulant (shipped overnight from Lonza, in a 50ml BD conical tube at room temperature, 1 M-125) in 1:1 D-PBS CTS™ (Invitrogen) was centrifuged at 800×*g* on a Histopaque®-1077 (Axis Shield) gradient to remove the red blood cells, followed by centrifugation of the resulting supernatant in 1:2 D-PBS CTS™ at 500×*g* to remove the platelets and plasma. The cells were resuspended in StemPro® MSC SFM CTS™ complete medium (Invitrogen) and cultured in T75 flasks (Corning). After 1 week, all attached cells were trypsinized (TrypLE™ Select CTS™, Invitrogen) and collected, followed by direct reprogramming into NPCs as follows: a synthesized polycistronic vector with an EF1-α promoter containing human Msi1, Ngn2, and MBD2 transcription factors linked by 2A peptides was introduced into the cells via nucleofection (4D Nucleofector™, Lonza). Transfection efficiency for each transfection was approximately 70% based on the expression of a separate GFP reporter vector included with the 4D nucleofection kit. Cells were cultured in a T75 flask (Corning) coated with human laminin (Millipore AG56P) in 5% CO_2_, 5% O_2_, and 37 °C in complete NeuroCult™-XF Proliferation medium (StemCell Technologies) supplemented with epidermal growth factor (EGF) [20 ng/ml] (CellGenix), fibroblast growth factor-2 (FGF-2) [30 ng/ml] (CellGenix), Valproic Acid [VPA, 1 mM] (Sigma-Aldrich), and Noggin [20 ng/ml] (R&D Systems). Cells were fed by replacing 50% of the medium every 36 h. The plasmid containing Msi1, Ngn2, and MBD2 was re-introduced into the cells after 2 days via lipofection (Lipofectamine® LTX & Plus™ Reagent, Invitrogen). VPA and Noggin were replaced by heparin [100 ng/ml] (Scientific Protein Laboratories) after 6 days of culture at the start of drNPC formation, and the cells were first passaged after 12 days in culture with StemPro® Accutase® (Invitrogen). The drNPCs continued to be expanded and passaged for additional 4 weeks until a total of one billion drNPCs were obtained and cryopreserved for all further studies. Thawed cells were cultured as a monolayer and were passaged 1:4 or 1:8 upon reaching < 80% confluency. The methodology used to generate BMC-derived drNPCs is identical to that used for human foreskin fibroblast (HFF)-derived and keratinocyte-derived drNPCs. HFFs and keratinocytes were purchased from ATCC (catalog #2097 and # 200-011, respectively).

### Maintenance and expansion of drNPCs

Human cells were cultured as a monolayer on Corning® CellBIND® culture dishes (Corning, Product #2394 to #3296) in low oxygen conditions in 5% CO_2_; 5% O_2_, and 37 °C. Complete Human NeuroCult XF medium (StemCell Technologies) was supplemented with epidermal growth factor (EGF) [20 ng/ml] (Peprotech), fibroblast growth factor-2 (FGF-2) [30 ng/ml] (Peprotech), and heparin [100 μg/ml] (Scientific Protein Laboratories). Accutase (Innovative Cell Technologies, Inc. Catalog #AT-104) was used for detaching the cells. Cells were passaged 1:4 or 1:8 upon reaching < 80% confluency. Cells were fed by replacing 50% of the medium every 36 h.

### Differentiation of drNPCs for IHC analysis

drNPCs were seeded onto laminin-coated (20 μg/ml; 100ul/well for 1 h at RT) Corning® CellBIND® 96 well plates. Cells were grown in differentiation media with the following composition: Neurobasal Media containing B27 (1X), BDNF (20 ng/ml), FGF2 (5 ng/ml), CNTF (20 ng/ml), PDGFaa (30 ng/ml), and T3 (30ng/ml). Media was changed after every 2–3 days until each plate was fixed for immunocytochemistry for checking markers for various cell types at early (1–3 days), mid (6–7 days), and late (14–16 days) timepoints.

### Differentiation of drNPCs for RT-qPCR analysis

Monolayers of drNPCs were plated onto adherent Corning® CellBIND® culture dishes (Corning, Product #2394 to #3296) in either maintenance culture medium (as mentioned above) or in the NeuroCult NS-A Differentiation Kit, comprising of NeuroCult XF basal medium (catalog # 05760) and NeuroCult™ NS-A Differentiation Supplement (Human) (Component# 0574) (StemCell Technologies). Media was changed after every 2–3 days until each plate was used for PCR analysis. The differentiation commenced for 10 days, and the differentiation profile was analyzed.

### drNPC sphere generation and sphere passaging

Monolayers of drNPCs were lifted off from adherent Corning® CellBIND® culture dishes (Corning, Product #2394 to #3296) using Accutase (Innovative Cell Technologies, Inc. Catalog #AT-104), and the resulting cell suspension was plated on Corning™ Costar™ 24-well Ultra-Low Attachment Surface Plates (Fisher Scientific, Product #07-200-602) at a 10 cells/μL density in the same medium used for culturing monolayers. After a 1 week incubation period, single primary spheres were dissociated into single cells and replated in fresh medium. Dissociation of cells consisted of suspending spheres in Accutase for 3 min at 37 °C and mechanically triturating the solution 20 times. The number of secondary spheres were counted in each well 1 week later.

### In vitro immunohistochemistry

For immunohistochemical analysis, cells were fixed with BD CytoFix fixation buffer (554655, BD Biosciences), permeabilized with phosphate buffer saline (PBS) containing 0.1% Triton-X100 and 3.7% formaldehyde, and blocked with PBS containing 5% normal goat serum (NGS), 0.01% Triton-X 100 and 0.1% Tween-20. Cells were then incubated overnight at 4 °C with primary antibodies diluted in PBS containing 1% NGS, 0.001% Triton-X, and 0.01% Tween-20: R-phycoerythrin (PE) coupled mouse anti-human Sox2 (562195, BD Biosciences); Alexa 647 coupled mouse anti-Nestin (560393, BD Biosciences); Alexa 488 coupled mouse monoclonal anti-human Pax6 (561664, BD Biosciences); Alexa 488 coupled mouse anti-βIII-tubulin (Class III, Tuj1 clone 560338, BD Biosciences); Alexa 647 coupled mouse anti-GFAP antibody (560298, BD Biosciences); mouse anti-Stro-1 (39-8401, ThermoFisher Scientific); Alexa 488 anti-NG2 (562413, BD Biosciences); Alexa 488 anti-ASCL1 (bs-1155R-A488, Bioss Antibodies); PE anti-Doublecortin (561505, BD Biosciences); Alexa 488 anti-Map2b (5650399, BD Biosciences); Alexa 488 anti-A2B5 (563776, BD Biosciences), and anti-human GFAP (STEM123) (Y40420, Takara). Preparations were incubated with 10 mg/mL Hoechst-33258 (Thermo Fisher Scientific) to visualize the nuclei. Images were acquired by widefield microscopy using the Cellomics High-Content Screening Arrayscan CX7 (Thermo Fisher Scientific) equipped with a dry 20x objective and the appropriate filters set (dichroic and emission filter: penta-band BGRFRN for 386/23, 521/22, 604/630, and 704/54 nm).

### FACS

For cytometry analysis, drNPCs were trypsinized and resuspended in Stain buffer (554657, BD Biosciences) before fixation in BD CytoFix buffer. Cells were permeabilized and washed in Perm/Wash buffer (554723, BD Biosciences) and incubated overnight in Stain buffer with antibodies for neuronal markers: DyeLight755 anti-nestin (NB300-266IR, Novusbio), V450 anti-Sox2 (561610, BD Biosciences), PE anti-Doublecortin, APC anti-CD133 (130-090-826, MACS Miltenyi Biotec), NL557 anti-βIII-tubulin (NL1195R, R&D Systems), and Alexa 488 anti-GFAP (560297, BD Biosciences). Images were acquired on FlowSight® Imaging Flow Cytometer (Amnis, Millipore), and data were analyzed using IDEAS® software (Amnis, Millipore).

### Karyotype analysis

For karyotyping, the culture medium was changed 24 h before harvesting drNPCs to stimulate cell division and increase mitotic index. When cells reached 80% confluency, Colcemid (10 μg/ml) was added 4 h before harvesting. The cells were harvested by centrifugation at 1000 rpm for 5 min and resuspended in 0.075 M pre-warmed KC1 (0.5%) for 10 min at 37 °C. The cells were then promptly centrifuged at 1000 rpm for 10 min and fixed in a freshly prepared 3:1 methanol/acetic acid solution for 30 min. The fixative was then changed three times. One or two drops of the cell suspension were placed on an alcohol clean slide that has been wetted in distilled water at room temperature and held at a 45° angle. The slides were immersed in absolute methanol for 10 min, quickly dried and stained with 1:80 dilution of Giemsa in 0.13 M phosphate buffer, pH 6.7 for 30 min. After staining, the slides were washed rapidly with tap water and air-dried. Monitoring of metaphase quality prior to staining was achieved by mounting the slide preparation in ethylene dichloride and observation of the major chromosome bands by Light microscopy (CellLine Genetics).

### PCR template preparation for positive control

Two PCR primers were designed to amplify a 4.5 kb sequence of the integration plasmid containing the recombinant genes. The PCR fragment was then gel purified, followed by column purification and quantification. To establish the standard curve, the positive control template was serially diluted in nuclease-free water to 10,000, 1000, 100, and 10 copies using the tool from the following website: http://cels.uri.edu/gsc/cndna.html. The primer sequences (5′–3′) are CCTCTTTACGGGTTATGG (sense) and AGTGCTACATTTCCAGTAG (antisense).

### Genomic extraction and purification of patient sample DNA

Approximately 10^6^ cells were used to extract genomic DNA for each sample using the Quick-gDNA MiniPrep kit from Zymo Research as per manufacturer instructions. Cells were lysed and pure genomic DNA was extracted, washed and column purified, followed by quantification. For multiplex qPCR, 10,000 copies of genomic DNA were used to detect integration. To calculate copy number for genomic DNA, the following website was used and each sample was diluted accordingly:https://www.thermofisher.com/ca/en/home/brands/thermo-scientific/molecular-biology/molecular-biology-learning-center/molecular-biology-resource-library/thermo-scientific-web-tools/dna-copy-number-calculator.html

### PCR primers and probes

Primers and dual-labeled probes were designed along the integration plasmid recombinant region using Oligoarchitect (Sigma). For a three-target multiplex PCR reaction, each probe was labeled with a different fluorophore on the 5′ end: FAM, HEX, or Cy5. All probes had the same dark quencher on the 3′ end. All primers/probes were resuspended in nuclease-free water to 100 μM. Working stocks were prepared by diluting primers and probes in nuclease-free water to 20 μM. Primer and probe sequences (5′–3′) used were as follows:

PCR1-sense: CTGTGCAAAGCGTTCATC,

PCR1-antisense: GCTTCTTCGAGCTTCTTG,

PCR-1-probe: CGCTCCTCCTGCTTCCTGAT.

PCR2-sense: GACCCTCTGAATCAGAAC,

PCR2-antisense: GTTGGTGACCTTAGTGAC,

PCR2-probe: CGGACCTGAACACCACCTTG.

PCR3-sense: CCGATATTCTGAGCAGAG,

PCR3-antisense: GCAGGCTGAAGTTAGTAG,

PCR3-probe: CTTCGTCGCCGCTATCCATT.

PCR4-sense: GCTGATGTTCGACAAGAC,

PCR4-antisense: GTGGAAATGGATCTCGCA,

PCR4-probe: TTCTCCACGATGTCCTCCGATTC.

Ubiquitous Promoter-sense: CCTCTTTACGGGTTATGG,

Ubiquitous Promoter-antisense: CTCGGGATCAAGAATCAC,

Ubiquitous Promotor-Probe: CTTGCGTGCCTTGAATTACTTCCA.

### Real-time PCR conditions

The mastermix used for these assays was the PrimeTime Gene Expression Mastermix (IDT, catalog # 1055770). Reagents were added to 96-well PCR plates from Applied Biosystems and briefly centrifuged before loading onto the qTOWER Real-Time PCR thermocycler (Analytik Jena). Cycling conditions were 3 min at 95 °C, 39 cycles of 5 s at 95 °C, 30 s at 60 °C followed by incubation at 65 °C.

### Absolute quantification

For absolute quantification analysis, standard curves were generated for primer pairs (above) via the serial dilutions of generated PCR templates. In accordance with MIQE (Minimum Information for Publication of Quantitative Real-Time PCR Experiments) guidelines, the amplification efficiencies (E) of reported runs were between 88% and 101% and *R*^2^ > 0.995. Copy numbers were derived from the standard curve formulas: PCR1: *y* = − 3.4266*x* + 37.801; PCR2: *y* = − 3.2892*x* + 38.053; PCR3: *y* = − 3.285*x* + 39.635; PCR4: *y* = − 3.3554*x* + 38.559. Ubiquitous promotor: *y* = − 3.647*x* + 39.24, where *x* denotes “log copy number” and *y* denotes the quantification cycle (Cq) value.

### Quantitative reverse transcription polymerase chain reaction (RT-qPCR) for gene expression

Samples were collected into Buffer RL (Norgen Biotek) and processed according to the manufacturer’s directions using Total RNA Purification Kit (Norgen Biotek—Catalog #17200). cDNA synthesis was carried out with iScript gDNA Clear cDNA Synthesis Kit (Bio-Rad—Catalog #1725034). cDNA quantity was measured using Qubit Detection System (Qubit DNA HS Assay Kit—Catalog # Q32851 and Qubit 4 Fluorometer). RT-qPCR reactions were prepared according to the manufacturer’s directions using SsoAdvanced Universal SYBR Green Supermix (Bio-Rad—Catalog# 172-5270) with normalized quantities of cDNA. RT-qPCR was carried out on Bio-Rad CFX384 Touch Real-Time PCR System (Bio-Rad). Cycling conditions consisted of polymerase activation and DNA denaturation (3 min at 98 °C), followed by 40 cycles of 10 s at 95 °C and 30 s at 60 °C. All reactions were concluded by incubation at 65 °C and increasing the temperature (at 0.5 °C increments) to 95 °C for melting-curve analysis. Relative expression analyses were carried out in accordance with MIQE (Minimum Information for Publication of Quantitative Real-Time PCR Experiments) guidelines, with minimum of two technical replicates per reaction. The Bio-Rad SYBR Green Assays used were Map2 (qHsaCIP0031486), Olig1 (qHsaCEP0025686), Gfap (qHsaCID0022307), and Gapdh (qHsaCED0038674). Relative expression data were normalized to the reference gene Gapdh to control for variability in expression levels and were analyzed using the Livak and Schmittgen (i.e., 2^−ΔΔCT^) method. The relative expression of each target was assessed by unpaired two-tailed *t* test. A *p* value of < 0.05 was considered significant.

### Surgical procedure for cell transplantation into the uninjured brain

Uninjured Fox Chase SCID/Beige (8–16 weeks old; CB17.Cg-Prkdc^scid^Lyst^bg-J^/Crl; Charles River Laboratories) and immunosuppressed Shiverer (Shi−/−) (8–weeks-old C3Fe.SWV-*Mbp*^*shi*^*/*J; The Jackson Laboratory) mice were anesthetized using isoflurane (1–2%). Shi−/− mice received 10 mg/kg/day of CsA (Bioshop Canada Inc., CYC002), dissolved in 65% ethanol: 35% Cremaphor vehicle, through osmotic mini-pumps (Alzet; 1002; 0.25 μl/hour) starting 3 days prior to transplantation and until sacrifice. The skull was exposed and a small hole was drilled using stereotaxic coordinates to permit injection of 1 × 10^5^ cells in a total volume of 1 μl through a Hamilton syringe (Hamilton). drNPCs were derived from monolayer cultures (passage 5–9) using Accutase [0.05 mL/cm^2^], collected, and suspended as single cells in artificial cerebrospinal fluid (aCSF). In SCID/Beige mice, cells were injected into the dorsolateral corner of the lateral ventricle (coordinates, relative to bregma; 0.3 mm anterior, 1.2 mm lateral, and 2.3 mm ventral from the surface of the skull). In Shi−/− mice cells were transplanted onto the corpus callosum (coordinates, relative to bregma; 1.0 mm anterior, 1.0 mm lateral, and 2.3 mm ventral from the surface of the skull). All experimental protocols were approved by the animal care committee of the University of Toronto in accordance with the policies established in the *Guide to the Care and Use of Experimental Animals* prepared by the Canadian Council of Animal Care.

### Immunohistochemistry and quantification for mouse brain sections

Mice were perfused transcardially with 1× phosphate-buffered saline (PBS) and 4% paraformaldehyde (PFA) in 1× PBS, followed by 6-h post-fixation in 4% PFA, and cryoprotection in 30% sucrose for at least 24 h at 4 °C. Coronal brain sections (20 μm) were cryosectioned and placed on Superfrost Plus Microscope Slides (Catalog #12-550-15, Fisherbrand). Samples were rehydrated for 5 min in PBS and permeabilized using 0.3% Triton in PBS for 20 min, followed by three washes with PBS. After, blocking solution containing 5% NGS and 1% BSA was applied to the samples for 1 h at room temperature. Next, the samples were incubated with primary antibodies overnight at 4 °C. Next day, samples were washed three times in 1× PBS and incubated with the secondary antibodies and nuclear stain at room temperature for 1 h. The samples were then mounted with Mowiol® 4-88 mounting media (Sigma, catalog # 81381) for imaging and long-term storage.

Sections were stained with the primary antibodies anti-human nuclei (HuNu) (Mouse monoclonal IgG1, 1:200, Millipore MAB1281), anti-human cytoplasm (STEM121) (Mouse monoclonal IgG1, 1:1000, Takara Y40410), anti-glial fibrillary acidic protein (GFAP) (Rabbit polyclonal IgG, 1:1000, Dako Z0334), anti-myelin basic protein (MBP) (Rat monoclonal IgG2a, 1:50, Abcam AB7349), anti-olig2 (Rabbit polyclonal IgG, 1:200, Millipore AB9610), anti-neuron specific enolase (NSE) (Mouse monoclonal, IgG2a, 1:250, Millipore MAB324), anti-neural cell adhesion molecule (NCAM) (Rabbit Polyclonal, IgG, 1:200,Abcam AB75813), and anti-Ki67 (Rabbit Monoclonal IgG, 1:200, Abcam AB16667). The following secondary antibodies were used: AlexaFluor 488 conjugated goat anti-rat (1:400, Invitrogen), AlexaFluor 568 conjugated goat anti-mouse (1:400, Invitrogen), AlexaFluor 647 conjugated goat anti-rabbit (1:400, Invitrogen), AlexaFluor 488 conjugated goat anti-rabbit (1:400, Invitrogen), and AlexaFluor 488 conjugated goat anti-mouse (1:400, Invitrogen). Tissue images were taken with ZEN Zeiss Spinning disk confocal microscope (ZEISS).

To quantify the overlapping expression of STEM121^+^/MBP^+^ and STEM121^+^/GFAP^+^ cells in Shi−/− brains, five 20× confocal stacks in three representative coronal sections per injection site were captured. The region of interest of the captured image was identified and a colocalization coefficient was calculated by delineating between non-colocalizing pixels, colocalizing pixels, and non-specific background pixels between each fluorescent channel. The relative number of colocalized GFAP or MBP pixels with STEM121 pixels for the five confocal stacks were averaged in each of the three representative sections and plotted as fold-change between 1 and 2 weeks post-transplant. A sample pixel co-localization analysis has been shown in Additional file 8: Figure S8.

To quantify HuNu^+^ and HuNu^+^/Ki67^+^ cells, the numbers of positive cells were counted in 20x confocal images from every 5th section extending from the rostral tip of the crossing of the corpus callosum to the anterior third ventricle and plotted as an average number per section.

### Electrophysiology

To generate neurons for analysis, drNPCs were cultured for 7 days on PLL/laminin substrate in neurobasal medium supplemented with B27, N2, FGF2 (10 ng/ml; Peprotech), EGF (10 ng/ml; Peprotech), and heparin (100 μg/ml; Scientific Protein Laboratories). On day 7, FGF and EGF were replaced with NT3 (10 ng/ml; Peprotech), BDNF (10 ng/ml; Peprotech), GDNF (10 ng/ml; Peprotech), RA (1 nM), cAMP (100 ng/ml), and ascorbic acid (0.1 mM). After 7 days, the cells were passaged to 24 well plates onto coverslips with human drNPC-derived astrocyte monolayers at a density of 8 × 10^4^ cells/well in neurobasal medium supplemented with B27, N2, BDNF (5 ng/ml), and GDNF (5 ng/ml) for another 4–6 weeks before being taken for patch clamp recordings. Co-culturing with human astrocytes has been shown to improve the functional maturation of human stem cell-derived neurons [40]. To generate the human astrocyte monolayer, drNPCs were cultured on Matrigel in DMEM/F12 supplemented with B27, 0.1% fetal bovine serum (FBS), BMP4 (10 ng/ml; Peprotech), and CNTF (5 ng/ml; Peprotech) for 14 days. Throughout the entire protocol, every 3 days half of the culture medium was replaced with fresh medium.

Whole-cell patch clamp recordings were performed on cells on a coverslip placed in a perfused chamber mounted on a Nikon E600FN microscope equipped with infrared differential interference contrast optics (IR-DIC). The extracellular solution contained (in mM): 140 NaCl, 5 KCl, 2 CaCl_2_, 1 MgCl_2_, 10 HEPES, 15 d-glucose, and pH adjusted to 7.2 with NaOH (osmolarity 292 mOsm). The patch pipettes were filled with a solution containing (in mM) 130 K-gluconate, 5 NaCl, 1 EGTA, 0.1 CaCl_2_, and 2 MgATP (pH 7.2 adjusted with KOH, osmolarity 260) and had resistances between 3 and 5 MΩ. All recordings were done at room temperature. The cells were approached with a patch pipette under visual control (IR-DIC, 40x water immersion objective). The signals, low-pass filtered at 10 kHz and sampled at 20 kHz, were recorded with a MultiClamp 700B amplifier, digitized with Digidata 1440 and processed/analyzed using pClamp10 software (all from Molecular Devices). The seal and cell quality were monitored using the Membrane Test function of the Clampex module of pClamp 10. Capacitance compensation and bridge balance were performed using the automatic functions of the MultiClamp 700B amplifier. The recorded voltages were corrected offline for 10 mV liquid junction potential. To record Na and K currents in voltage-clamp mode, the cells were held at − 80 mV, and voltage steps were applied with 10 mV increments between − 110 mV and + 40 mV. To test the ability of cells to generate action potentials in current clamp mode, the membrane potential was held near − 70 mV, and 50 and 250 ms current steps were applied to depolarize the membrane to action potential threshold levels, and up to 0 mV or higher when no action potentials were detected.

## Results

### Induction and in vitro characterization of drNPCs

**Fig. 1:**
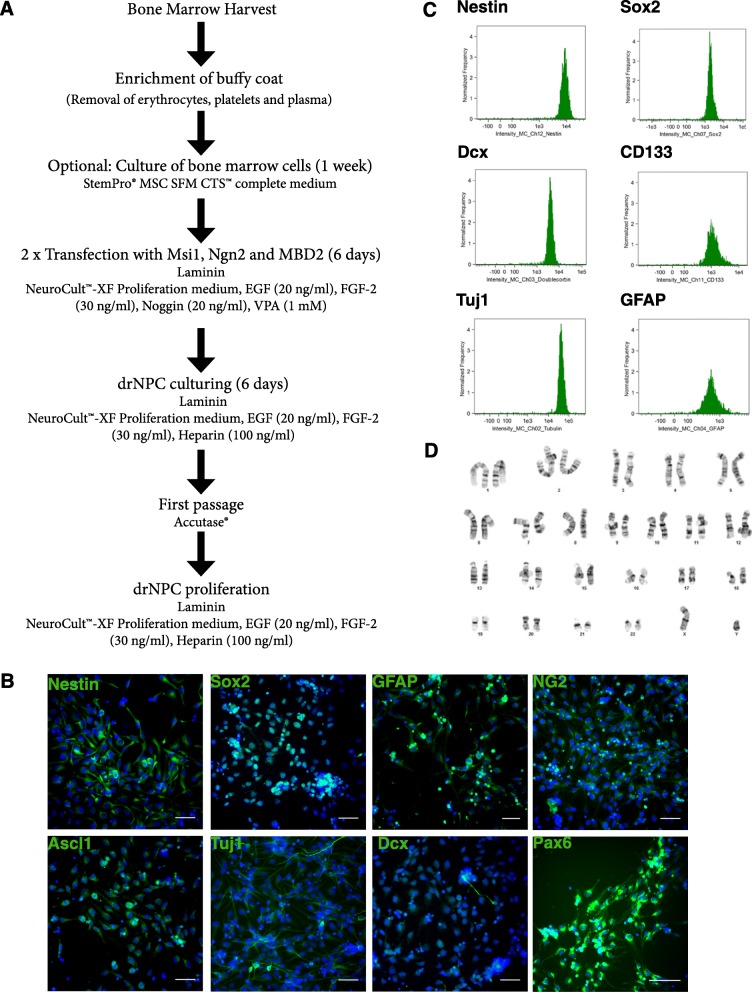
Generation and in vitro characterization of drNPCs. **a** Schematic of protocol for reprogramming cells to drNPCs. **b** At passage 7, drNPCs continue expressing high levels of Nestin, Sox2, GFAP, NG2, Ascl1, Tuj1, Dcx, and Pax6. Nuclei stained with Hoechst (blue). Scale bar = 100 μm. **c** drNPCs at passage 12 were analyzed for expression of multiple markers, graphed by intensity of expression to determine cell population purity, using a 12-channel Amnis FlowSight® Imaging flow cytometer. **d** Normal karyotype of drNPCs at passage 12

Herein, drNPCs were generated by transiently introducing Msi1, Ngn2, and MBD2 into bone marrow-derived cells (BMCs), human foreskin fibroblasts (HFFs), and keratinocytes for 6 days. Using this reprogramming method, we were able to obtain a neural precursor cell phenotype from these different starting cell populations, as shown by Nestin, Sox2, and GFAP expression (Fig. 1, Additional file 1: Figure S1) within 2 weeks. Furthermore, drNPCs cultured in neural stem cell conditions post-transfection could be continually passaged and propagated in the same conditions (Fig. 1a) for at least 26 passages (the longest time examined). drNPCs express NPC markers (Nestin, Sox2, Pax6), neuronal markers (Tuj1, Dcx, Ascl1), and glial markers (NG2, GFAP) (Fig. 1b) and flow cytometry further revealed a robust expression of Nestin, Sox2, Dcx, CD133, Tuj1, and GFAP (Fig. 1c). drNPC karyotype remained normal for up to 12 passages (the longest time examined) (Fig. 1d). Quantitative reverse transcription PCR (RT-qPCR) demonstrates a lack of gene integration of the plasmid into the reprogrammed drNPCs (Additional file 2: Figure S2).

**Fig. 2:**
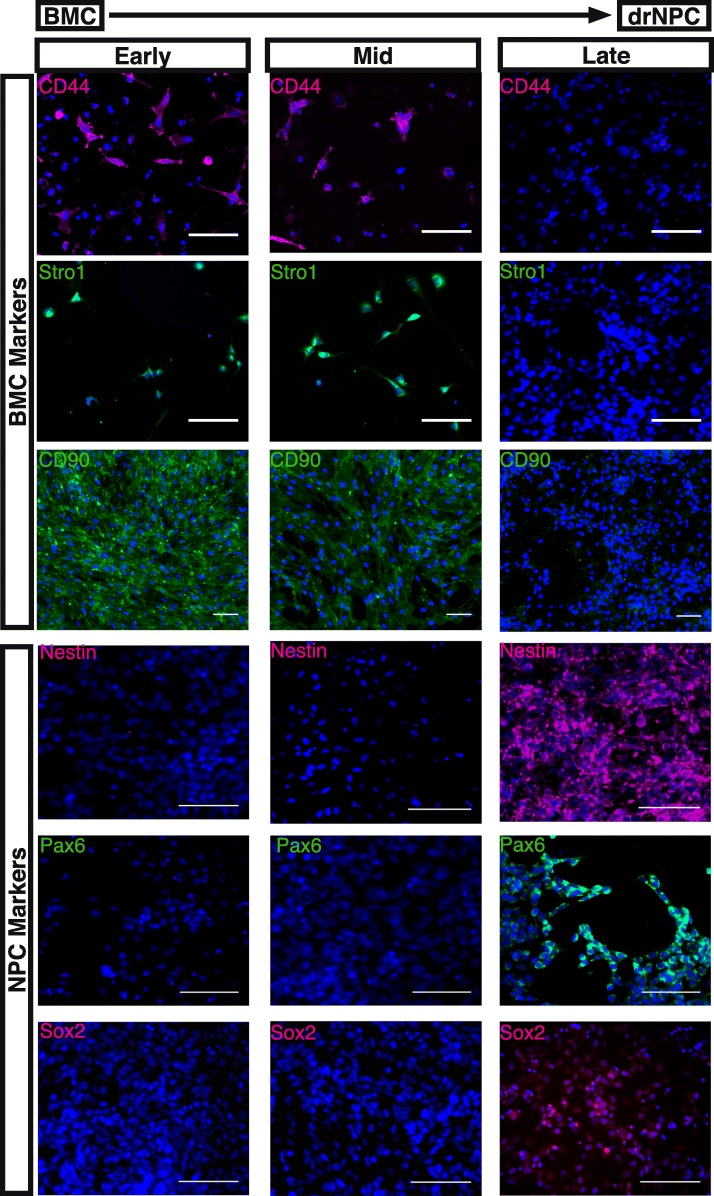
BMC to drNPC reprogramming. In vitro cultures of bone marrow cells (BMCs) during reprogramming to drNPCs. At early time-points (days 1–3 in vitro), BMC-specific markers CD44, Stro1, and CD90 are expressed and no NPC-specific markers Nestin, Pax6, or Sox2 are observed. By the mid time-points (days 6–7 in vitro), downregulation of BMC-specific markers occurs. At late time-points (days 14–16 in vitro), no BMC-specific markers are observed and NPC-specific markers Nestin, Pax6, and Sox2 are highly expressed. Nuclei stained with Hoechst (blue). Scale bar = 100 μm.

**Fig. 3:**
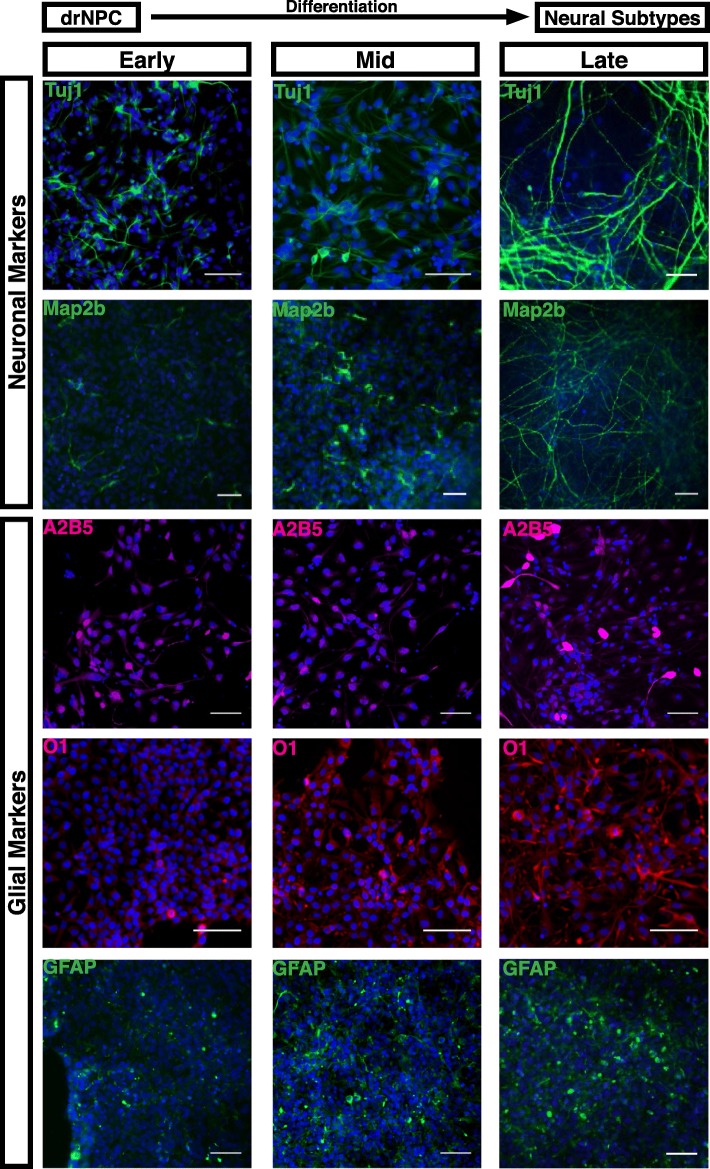
drNPC differentiation in vitro. In differentiation conditions, drNPC monolayers give rise to neurons (Tuj1 and Map 2b), oligodendrocytes (A2B5 and O1) and astrocytes (GFAP). Early = 1–3 in vitro; mid = days 6–7 in vitro; late = days 14–16 in vitro. Nuclei stained with Hoechst (blue). Scale bar = 100 μm.

In terms of reprogramming efficiency, 100% of the trials performed with BMC and HFF starting populations (> 20 per cell source) generated directly reprogrammed NPCs (shown in Fig. 2 by the expression of neural precursor markers Nestin and Sox2). The reprogramming efficiency was characterized using immunohistochemistry, by quantifying the percentage of Sox2 expressing cells at 12 days post-transfection. Sox2 expression ranged from 50 to 98% in all wells examined across all trails (72 ± 8.97% and 86.25 ± 7.23% of cells expressing Sox2 in reprogrammed BMCs and HFFs, respectively, *n* = 8 independent trials). These findings reveal that BMCs and HFFs have equivalent reprogramming efficiencies using this reprogramming strategy. With regard to BMC-derived drNPCs, immunohistochemistry during the reprogramming phase revealed a concomitant loss of BMC marker expression for CD44, Stro1, and CD90 and acquisition of NPC markers Nestin, Pax6, and Sox2 (Fig. 2). Furthermore, directed differentiation of drNPCs in neural differentiation medium resulted in a progressive increase in the expression of neuronal markers Tuj1 and Map2b, as well as glial markers A2B5, O1, and GFAP, as revealed by immunostaining (Fig. 3). In addition, RT-qPCR analysis comparing the expression levels of neural markers Map2, Olig1, and Gfap in drNPCs cultured in maintenance medium and drNPCs differentiated for 10 days revealed similar expression of Olig1, and increased expression of Map2 and Gfap, in differentiated drNPCs (Additional file 3: Figure S3). The IHC data is further corroborated by our BMCs that were placed in the same neural stem cell culturing conditions, in the absence of reprogramming factors these cells did not lose the expression of BMC markers CD44 and Stro1 and NPC markers Nestin, Pax6, and Sox2 were undetectable by immunostaining, confirming the absence of NPCs in the starting cell population (Additional file 4: Figure S4). Notably, the pluripotency markers Nanog, Oct4, SSEA-1, and TRA1-81 were never observed during the reprogramming phase (Additional file 5: Figure S5). Hence, the reprogramming strategy resulted in a robust production of integration-free NPCs without passing through a pluripotency state.

To evaluate whether the drNPCs exhibited neural stem cell characteristics, we employed the neurosphere assay. When drNPCs derived from monolayer cultures were plated at clonal density [41], they formed free-floating spheres in 97% of all plated wells (average of 1.39 ± 0.13 primary neurospheres per 5000 drNPCs plated, the average diameter of 217.37 ± 16.85 μm from 109 neurospheres across 3 independent experiments). Individual neurospheres dissociated and replated as single cells gave rise to secondary spheres (2.40 ± 1.05 secondary neurospheres/individual primary neurosphere) and gave rise to neurons, astrocytes, and oligodendrocytes, thus demonstrating self-renewal and multipotency (Additional file 6: Figure S6).

**Fig. 4:**
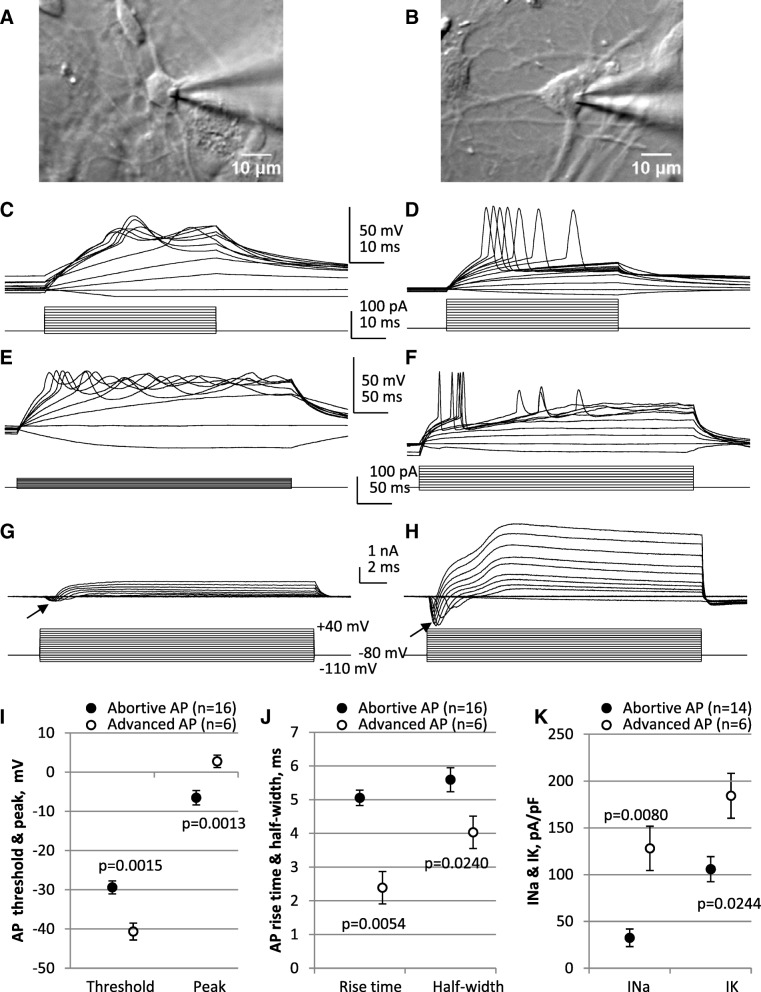
Electrophysiological profiles of neurons derived from drNPCs. **a**, **b** Infrared DIC images of the drNPCs, with patch pipette attached. **c**–**h** Whole cell recordings from cells shown in **a** and **b**. **c**–**f** Current clamp recordings of action potentials evoked with 50 ms (**c**, **d**) and 250 ms (**e**, **f**) current pulses of varying intensities. The current pulses are shown in lower traces of **c**–**f**. Note the differences between the sizes and shapes of abortive (**c**, **e**) and more advanced action potentials (**d**, **f**). **g**, **h** Voltage clamp recordings from these cells show smaller Na currents (downward deflections, shown with arrows) in the cell with an abortive action potential (**g**) compared to the cell with a more advanced action potential (**h**). The voltage steps from − 80 mV holding level, with 10 mV increments, are shown in lower traces. **i**–**k** Statistical comparison of action potential parameters and Na and K currents in cells with abortive and more advanced action potentials

We next performed electrophysiology on drNPC-derived neurons (Fig. 4). Action potentials were generated in response to depolarizing current steps by 50% of the neurons tested (22/44). Of these, ~ 27% (6/22) exhibited mature-like action potentials (Fig. 4d, e) while ~ 73% (16/22) had much broader and smaller (“abortive”) action potentials (Fig. 4c, e) [42]. Cells that generated action potentials exhibited inward Na^+^ currents in response to depolarizing voltage steps (Fig. 4g, h), with the Na^+^ currents being significantly smaller in amplitude in cells with abortive action potentials compared to cells with more advanced action potentials (Fig. 4i–k). The action potentials and inward currents were sensitive to the Na^+^ channel blocker tetrodotoxin (1 μM). These findings reveal that neurons from drNPCs are electrophysiologically functional.

### drNPCs respond to endogenous cues in vivo following transplantation into the uninjured brain

**Fig. 5:**
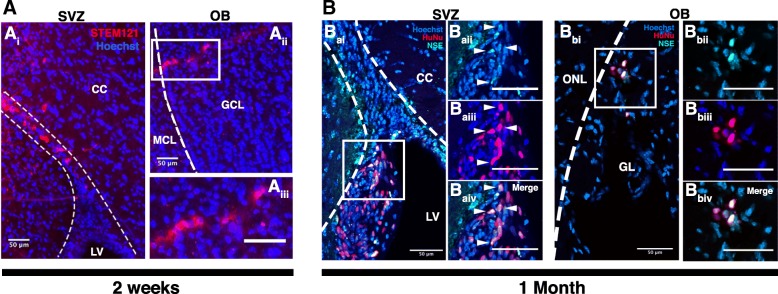
Transplanted drNPCs respond to migratory cues in vivo. **A** At 2 weeks post-transplant STEM121^+^ drNPCs are seen in both the anterior SVZ, near the site of transplant (**A**_**i**_), and in the OB (**A**_**ii**_, **A**_**iii**_). **B** At 1 month post-transplant HuNu^+^ drNPCs are found in both the anterior SVZ, near the site of transplant (**B**_**ai**_–**B**_**aiv**_), and in the OB (**B**_**bi**_–**B**_**biv**_). Arrowheads indicate HuNu^+^/NSE^+^ cells; white boxes outline higher magnification insets; CC corpus callosum, LV lateral ventricle, GCL granule cell layer, MCL mitral cell layer, ONL olfactory nerve layer, GL glomerular layer; scale bars = 50 μm. *n* = 3 transplanted hemispheres

We examined the in vivo behavior of transplanted drNPCs in two mouse strains: immunocompromised SCID/Beige mice and Shiverer mice (Shi^−/−^) immunosuppressed with Cyclosporine-A. In the first series of experiments, we transplanted drNPCs onto the rostral dorsolateral corner of the lateral ventricle subependyma of uninjured adult SCID/Beige mice. In mice sacrificed at 2 weeks or 1 month post-transplantation, we observed STEM121^+^ and HuNu^+^ (cytoplasmic and nuclear human-specific antibodies, respectively) drNPCs at the site of injection and in the olfactory bulb (Fig. 5A, B). At 1 month post-transplant, drNPCs at the site of injection and in the olfactory bulb expressed the neuronal markers neuron-specific enolase (NSE) (Fig. 5B) and neural cell adhesion molecule (NCAM) (Additional file 7: Figure S7), which is characteristic of endogenous NPCs [43–45]. At 1 month post-transplant, we observed 270 *±* 8.67 HuNu^+^ drNPCs in the olfactory bulb (representing 0.3% of the transplanted cells). In the olfactory bulb, 37.40 *±* 6.25% of HuNu^+^ cells were positive for NCAM. Hence, similar to transplanted murine NPCs, drNPCs survive and respond to migratory cues in vivo.

**Fig. 6:**
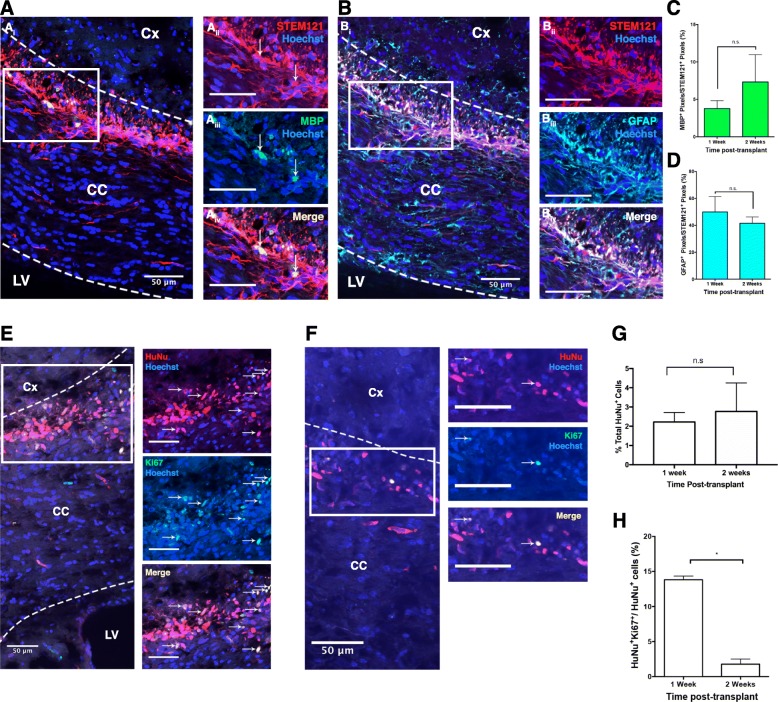
drNPC transplants in myelin-deficient Shiverer mice. drNPCs give rise to MBP and GFAP expressing cells post-transplant (**A**, **B**). STEM121 (red) expressing drNPCs express MBP (**A**_**iii**_; green) and GFAP (**B**_**ii**i_; turquoise) in the CC (outlined with dotted lines). **C**, **D** MBP and GFAP expression in the transplanted drNPCs between 1 week and 2 weeks post-transplant. **E**, **F** A subpopulation of transplanted drNPCs are HuNu^+^/Ki67^+^ on the corpus callosum of Shi^−/−^ mice at 1 week (**A**) and 2 weeks (**B**) post-transplant. White arrows indicate Ki67^+^/HuNu^+^ cells. There is no significant difference between the total number of HuNu^+^ drNPCs at the site of the transplant between 1 and 2 weeks post-transplant (**G**). There is a significant decrease in the percentage of Ki67^+^/HuNu^+^ drNPCs between 1 and 2 weeks post-transplant (**H**). Dotted lines indicate the border of corpus callosum border. Cx cortex, CC corpus callosum, LV lateral ventricle; scale bars = 50 μm, *n* = 4 transplanted hemispheres per group, * = *p* < 0.05; n.s. not significant

To further examine drNPC lineage potential, we transplanted cells into the corpus callosum of Shiverer (Shi^−/−^) mutant mice that do not express myelin basic protein (MBP). Mice were sacrificed at 1 and 2 weeks post-transplant. At both survival times, we observed MBP^+^/STEM121^+^ cells in the corpus callosum (3.8 ± 1.1% vs 7.3 ± 3.7% MBP^+^/STEM121^+^ pixels; 1 vs 2 weeks, respectively) (Fig. 6 A, C, D) demonstrating drNPC differentiation into oligodendrocytes, consistent with previous reports transplanting murine NPCs [44]. In addition, a subpopulation of STEM121^+^ cells expressed GFAP, indicating an astrocyte phenotype (50.1 ± 5.7% vs 41.6 ± 2.3% GFAP^+^/STEM121^+^ pixels; 1 vs 2 weeks, respectively) (Fig. 6B). We further assessed drNPC survival and proliferation by staining for HuNu and Ki67 (Fig. 6E-G). While the numbers of surviving transplanted cells (HuNu^+^) remained constant from 1 to 2 weeks post-transplant (2.22 ± 0.25% and 2.77 ± 0.74% of transplanted drNPCs survive, respectively) (Fig. 6G), the numbers of proliferating cells significantly decreased over this same period (13.8 ± 0.5% vs 1.8 ± 0.7%; 1 vs 2 weeks, respectively) (*p* < 0.05) (Fig. 6H). This degree of proliferation was similar to what was observed in SCID/Beige transplanted mice with 1.1% of drNPCs expressing Ki67 at 2 weeks post-transplant. Taken together, these transplant findings reveal that drNPCs can respond to endogenous cues for migration and differentiation following transplantation.

## Discussion

The acquisition of a neural phenotype via reprogramming can be accomplished by either inducing a pluripotent phase followed by differentiation towards the neural lineage [25, 26, 46–48] or by directly reprogramming cells towards either an NPC-like [16, 20, 32] or mature neuronal phenotype [28]. Herein, we have demonstrated the methodology of efficiently and directly reprogramming somatic cells to a neural precursor state via the transient expression of Msi1, Ngn2, and MBD2 for 1 week without the use of viral or integrating plasmids. Cell reprogramming has been previously described and the use of hiPSCs for regenerative therapies to treat macular degeneration and cortical stroke have been reported [22, 49]. Our approach introduces several novel features that provide a number of advantages. The transformation occurs without passing through a pluripotent state, thereby reducing risks associated with unintended teratoma formation. This is supported by our in vitro analysis that revealed no expression of pluripotency markers such as Oct4 and Nanog at any point during the reprogramming, along with transplantation studies in uninjured mice showing no evidence of tumor formation and significant downregulation of proliferation. In addition, the efficiency of this reprogramming paradigm, which allows for the production of more cells in less time, has important implications for clinical translation as the wait time for conventional reprogramming methods can be too long for use in the clinic [21]. Although our direct reprogramming methodology is not the only non-viral methodology to generate neural precursor cells within weeks [50, 51], we have demonstrated the generation of human NPCs at higher efficiencies and in larger quantities than previously reported. Both Wang and et al. and Capetian et al. reported an average reprogramming efficiencies around 0.2–0.4% per cell [50, 51]. Hence, we believe our reprogramming methodology is a feasible, time, and cost-effective methodology to be potentially employed in clinical settings. Lastly, drNPCs overcome the ethical issues associated with fetal and embryonic cell sources, as well as immunological concerns seen with non-autologous cell sources.

Our results demonstrate that using the transcription factors Msi1, Ngn2, and MBD2 to reprogram cells results in a cell population exhibiting a neural precursor phenotype. Many cells that have been reprogrammed from a different lineage require donor program repression, meaning they must be cultured with specific factors to prevent a reversal to the original cell phenotype [52, 53]. However, culturing drNPCs only requires EGF and bFGF, which are considered to be the minimal growth factor requirements for NPC culturing [4, 54]. This provides evidence that the reprogramming method results in a permanently reprogrammed population of cells within the neural lineage with no risk of reverting back to its previous cell state. In this study, drNPCs were propagated as a monolayer to passage 26 to assess the stem-like properties of the reprogrammed cells (self-renewal and multipotentiality). Given the highly proliferative nature of the drNPC monolayers, sufficient numbers of cells can be generated with far fewer passages for use in clinical settings. Importantly, we have shown drNPCs to be neurally committed, exhibit a neuronal electrophysiological profile, and be responsive to in vivo environmental and migratory cues. Although we characterized the drNPC neural phenotype under baseline conditions, it is noteworthy that the drNPC’s neural differentiation profile is dependent on the specific differentiation protocols (in vitro) and the microenvironment (when transplanted in vivo). In this manuscript, we demonstrated that drNPC-derived neurons generate action potentials only when cultured on drNPC-derived astrocytic monolayers. We believe this correlation, combined with the expression of GFAP in both of our in vitro and in vivo experiments, is evidence of the generation of mature astrocytes from drNPCs. Although our electrophysiological recordings have confirmed a neuronal phenotype in a population of drNPC-derived neurons as defined by the presence of sharp, overshooting, all-or-nothing, mature-like action potentials and large, fast, voltage-activated inward Na currents, none of the cells exhibited repetitive full-size action potentials that have been reported in cultured NPCs at later stages of development [42]. Further studies looking at longer times in vitro (for example) are warranted.

The Shiverer mice provide a model to enable the detection of MBP-expressing oligodendrocytes derived from transplanted drNPCs as the Shiverer mice do not express endogenous MBP. In addition, transplantation of drNPCs into the injured spinal cord has revealed drNPC-derived mature, compact myelin formation using electron microscopy which correlated with improved functional outcomes [55, 56] in the absence of tumor formation [56]. In addition, Vonderwalde, Azimi, et al. demonstrated the beneficial effect of drNPCs in a model of focal ischemia [57]. Collectively, these findings point to the potential utility of drNPCs in cell-based therapies for CNS insults. The immature precursor-like phenotype of drNPCs provides an edge over mature cell sources because neural precursor cells have higher viability after transplant in comparison to their more mature counterparts [58] and greater plasticity to differentiate into various neural and glial subtypes.

The utility of drNPCs and the described reprogramming method is not limited to therapeutic interventions. Indeed, reprogrammed cells can also be used to study patient-specific CNS diseases, opening up an avenue to use drNPCs to for drug screening or improve personalized medicine approaches or perform pre-clinical testing in vitro [12, 13, 59, 60].

## Conclusions

DrNPCs are a neurally committed cell source that can be generated with high efficiency. drNPCs provide an avenue for generating patient-specific cells, bypassing limitations associated with ethical, safety, and practical hurdles of acquiring NPCs that cell-based strategies often face when considering the optimal cell type candidates for transplantation in human therapeutic interventions. Further studies are needed to assess the efficacy of transplanting these cells in neurodegenerative diseases and other CNS injury models. Thus, drNPCs could hold significant promise for clinical applications to repair CNS-related insults and in neurodegenerative diseases.

## Additional files

Additional file 1: **Figure S1.** drNPCs can be generated from multiple starting cell populations. In addition to BMCs, drNPCs can be generated from other cell sources such human foreskin fibroblasts (HFF) and keratinocytes. The expression of neural markers Nestin, Sox2, and GFAP is only observed post-transfection (post-reprogramming). Scale bar = 100µm. (PDF 365 kb)

Additional file 2: **Figure S2.** The synthetic plasmid used in reprogramming does not integrate into drNPCs. RT-qPCR analysis demonstrates the lack of any plasmid sequences in reprogrammed drNPCs. 4 different primer pairs were designed to detect plasmid sequence (PCRs 1-4) and 1 ubiquitous promotor primer pair was used as positive control. Samples studied were control template (determined to be ~ 10 copy numbers), untransfected BMCs, and 3 distinct samples of drNPCs (drNPC1-3) made from different starting cells. UD = undetected. (PDF 137 kb)

Additional file 3: **Figure S3.** drNPC differentiation in vitro. Differentiated drNPCs express increased levels of Map2 and Gfap mRNA compared to control drNPCs that were cultured in maintenance media. There was no change in Olig1 expression. Data are shown as mean ± SEM. *n* = 3 biological samples per cohort. Gene expression levels are relative to control drNPCs and normalized to the reference gene Gapdh. (PDF 111 kb)

Additional file 4: **Figure S4.** BMCs do not acquire NPC characteristic when placed in the neural stem cell culturing conditions in the absence of reprogramming. BMCs placed in the neural stem cell culturing conditions maintain expression of BMC-specific marker CD44 and Stro1 and do not acquire any NPC marker expression (Nestin, Pax6, Sox2). Early = 1–3 in vitro; mid = days 6–7 in vitro; late = days 14–16 in vitro. Nuclei stained with Hoechst (blue). Scale bar = 100 μm. (PDF 496 kb)

Additional file 5: **Figure S5.** Pluripotency markers are not expressed during reprogramming. BMCs reprogrammed to drNPCs were analyzed for pluripotency markers Nanog, Oct4, SSEA-1, and TRA1–81 during the reprogramming process over time. No expression of pluripotency markers was observed. Early = 1–3 in vitro; mid = days 6–7 in vitro; late = days 14–16 in vitro. Nuclei stained with Hoechst (blue). Scale bar = 100 μm. (PDF 562 kb)

Additional file 6: **Figure S6.** drNPC-derived spheres can be derived from monolayers and give rise to neurons and glia in vitro. Dissociating 80% confluent drNPC monolayers and plating them at 10 cells/μl density results in free-floating spheres within 7 days of culturing. Differentiating spheres for additional 7 days in the presence of serum will result in GFAP-positive astrocytes, Olig2-positive oligodendrocytes, and Tuj1-positive neurons. Scale bar = 100 μm. Representative bright field images of drNPC monolayer (left) and spheres (centre), and a representative IHC image of differentiated drNPC-derived spheres. (PDF 249 kb)

Additional file 7: **Figure S7.** drNPCs present in olfactory bulb express markers of neuronal differentiation. drNPCs transplanted into the SCID/Beige animals are present in the olfactory bulb at 1 month post-transplant and contain a subpopulation of NCAM-positive cells (white arrows). Scale bars = 10 μm. (PDF 831 kb)

Additional file 8: **Figure S8.** Pixel colocalization analysis of cytoplasmic immunofluorescent staining. A: Co-localization measurement of cytoplasmic immunofluorescent staining in mouse brain cryosections. Zeiss Zen software was used to measure channel intensities of each immunofluorescently stained pixel and quantify a double positive pixel ratio in defined regions of interest. Threshold pixel intensities were adjusted first to non-labeled areas (Box 1), followed by fluorophore labeled secondary antibody backgrounds (Box 2) in the same section. Pixels of human cytoplasmic epitope-specific STEM121-labeled area (Box 3) was compared to STEM121-negative areas. The sample image, scatter plot of all three regions (Boxes 1, 2, and 3), and pixel measurements/co-localization coefficient are shown. (PDF 371 kb)

## Data Availability

The data that support the findings of this study are included in this published article. The drNPCs are being commercialized by Fortuna Fix Ltd., who provided the drNPC for the current study. The cells can be made available upon request under Fortuna’s MTA.

## Abbreviations

BMC: Bone marrow-derived cell
CNS: Central nervous system
drNPC: Directly reprogrammed neural precursor cell
FACS: Fluorescence-activated cell sorting
HFF: Human foreskin fibroblast
iPSC: Induced pluripotent stem cell
MBD2: Methyl-CpG-Binding Domain 2
MBP: Myelin basic protein
MSI1: Musashi1
NGN2: Neurogenin2
NPC: Neural precursor cell
RMS: Rostral migratory stream
RT-qPCR: Quantitative reverse transcription polymerase chain reaction
