# Supplementary figures and images for: Examining the fundamental biology of a novel population of directly reprogrammed human neural precursor cells
Source: Stem Cell Res Ther. 2019 Jun 13;10:166. doi: 10.1186/s13287-019-1255-4 (PMC6567617; doi:10.1186/s13287-019-1255-4)

Supplementary Figure 1

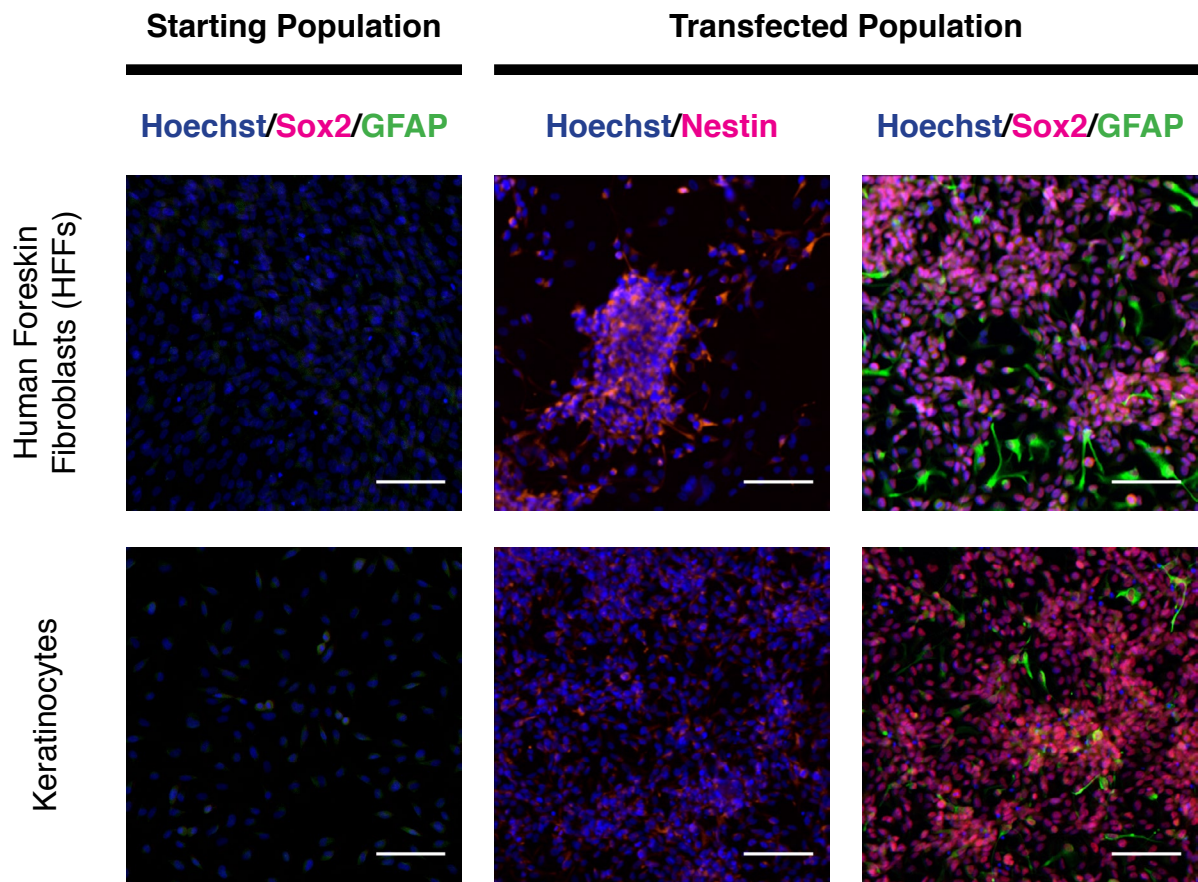

Supplement: Supplementary file 1 — Figure S1. drNPCs can be generated from multiple starting cell populations. In addition to BMCs, drNPCs can be generated from other cell sources such human foreskin fibroblasts (HFF) and keratinocytes. The expression of neural markers Nestin, Sox2, and GFAP is only observed post-transfection (post-reprogramming). Scale bar = 100µm. (PDF 365 kb) [file 13287_2019_1255_MOESM1_ESM.pdf]

Supplementary Figure 2

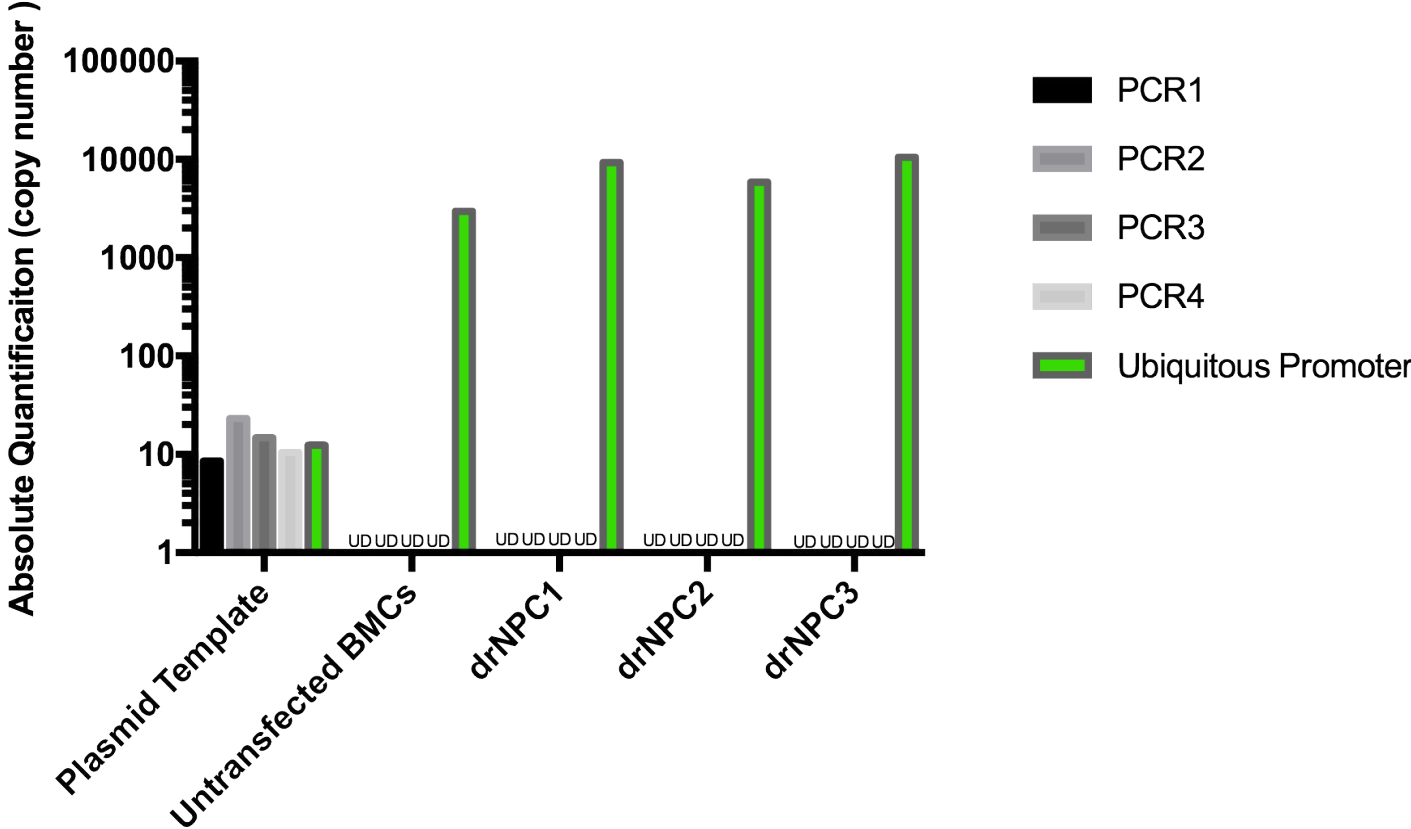

Supplement: Supplementary file 2 — Figure S2. The synthetic plasmid used in reprogramming does not integrate into drNPCs. RT-qPCR analysis demonstrates the lack of any plasmid sequences in reprogrammed drNPCs. 4 different primer pairs were designed to detect plasmid sequence (PCRs 1-4) and 1 ubiquitous promotor primer pair was used as positive control. Samples studied were control template (determined to be ~ 10 copy numbers), untransfected BMCs, and 3 distinct samples of drNPCs (drNPC1-3) made from different starting cells. UD = undetected. (PDF 137 kb) [file 13287_2019_1255_MOESM2_ESM.pdf]

Supplementary Figure 3

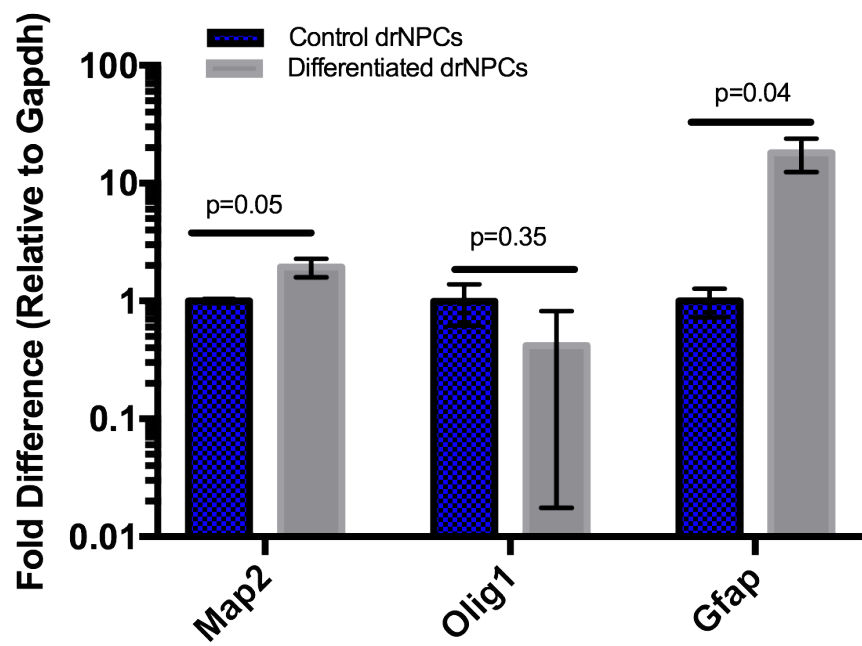

Supplement: Supplementary file 3 — Figure S3. drNPC differentiation in vitro. Differentiated drNPCs express increased levels of Map2 and Gfap mRNA compared to control drNPCs that were cultured in maintenance media. There was no change in Olig1 expression. Data are shown as mean ± SEM. n = 3 biological samples per cohort. Gene expression levels are relative to control drNPCs and normalized to the reference gene Gapdh. (PDF 111 kb) [file 13287_2019_1255_MOESM3_ESM.pdf]

Supplementary Figure 4

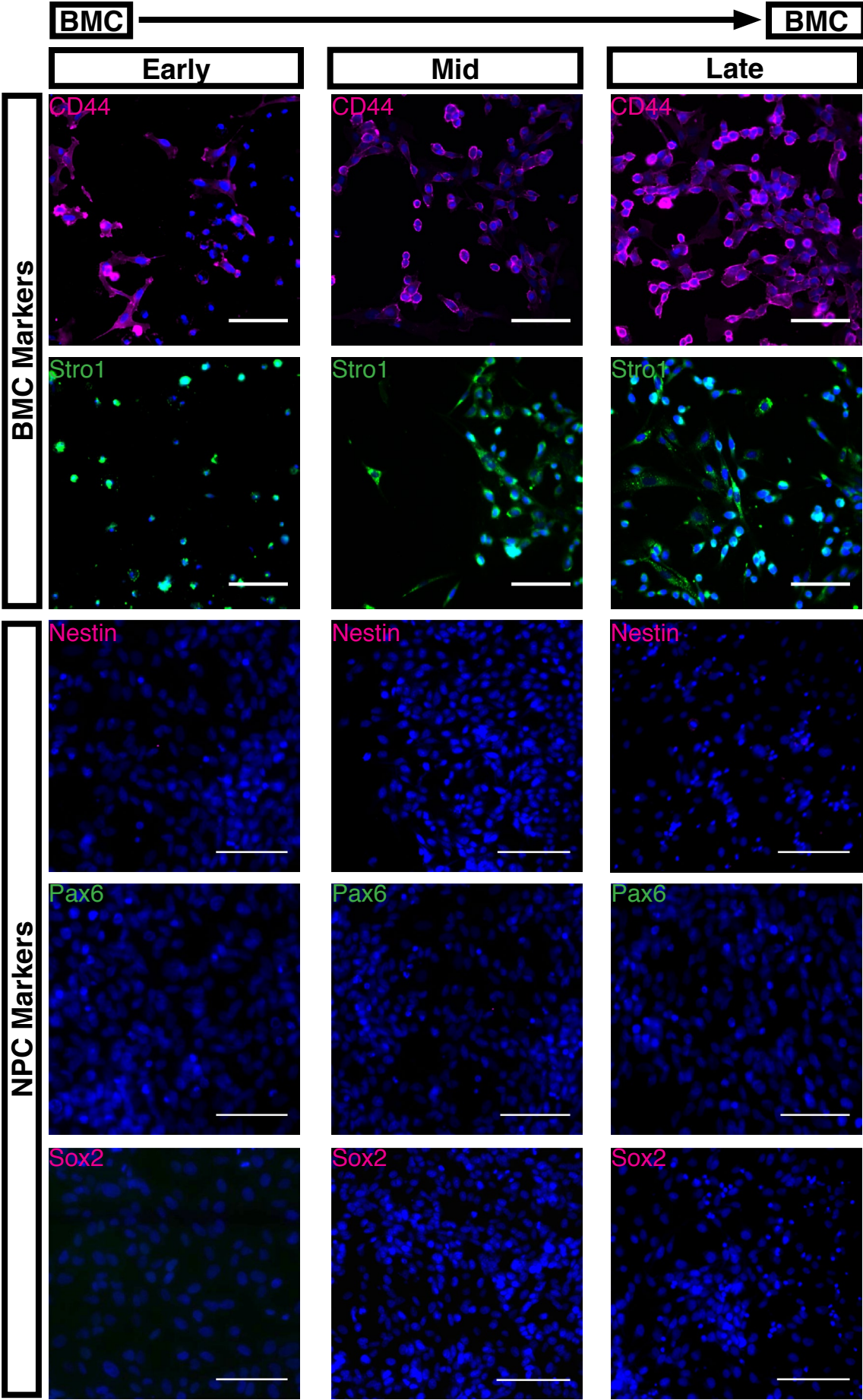

Supplement: Supplementary file 4 — Figure S4. BMCs do not acquire NPC characteristic when placed in the neural stem cell culturing conditions in the absence of reprogramming. BMCs placed in the neural stem cell culturing conditions maintain expression of BMC-specific marker CD44 and Stro1 and do not acquire any NPC marker expression (Nestin, Pax6, Sox2). Early = 1–3 in vitro; mid = days 6–7 in vitro; late = days 14–16 in vitro. Nuclei stained with Hoechst (blue). Scale bar = 100 μm. (PDF 496 kb) [file 13287_2019_1255_MOESM4_ESM.pdf]

Supplementary Figure 5

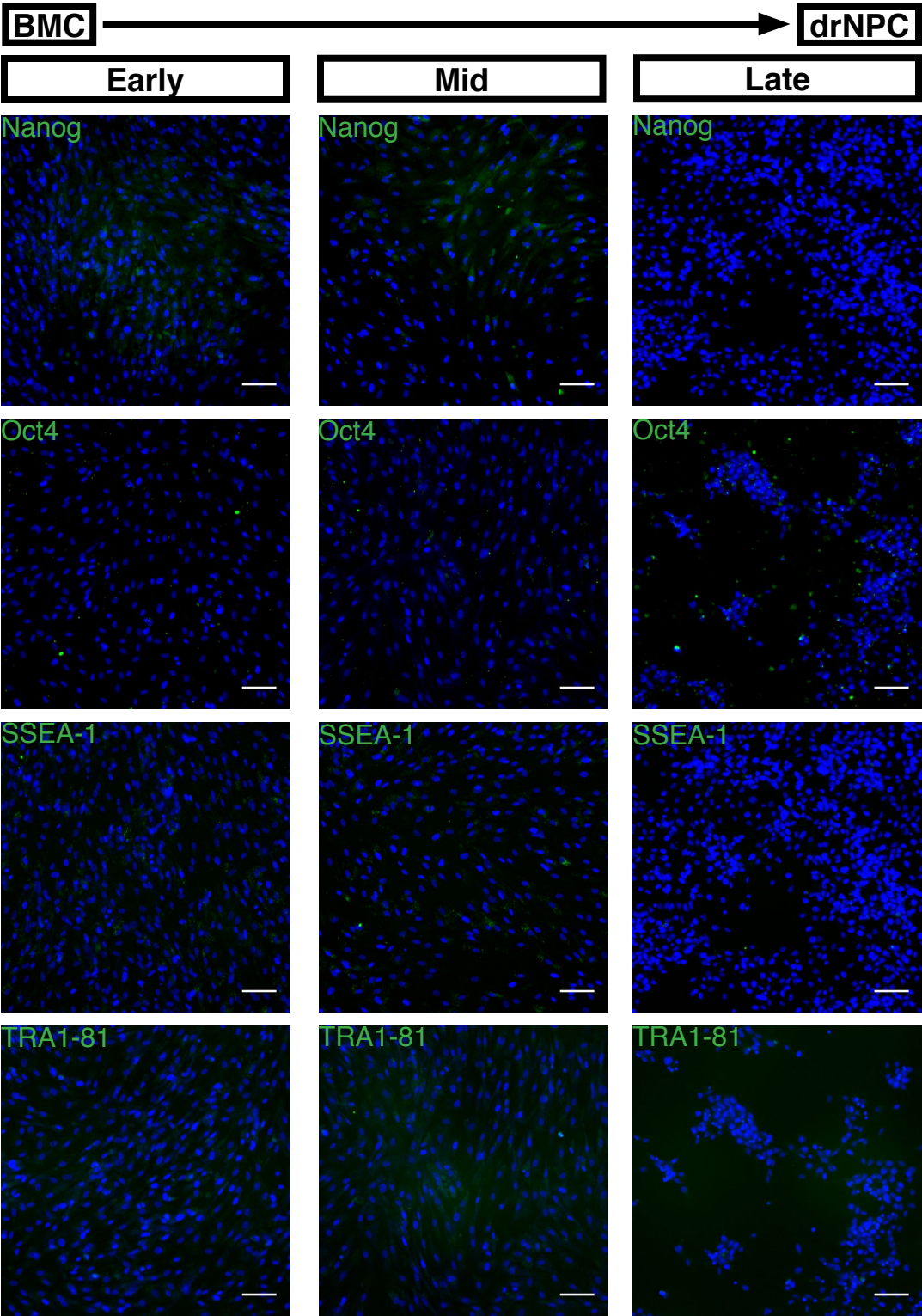

Supplement: Supplementary file 5 — Figure S5. Pluripotency markers are not expressed during reprogramming. BMCs reprogrammed to drNPCs were analyzed for pluripotency markers Nanog, Oct4, SSEA-1, and TRA1–81 during the reprogramming process over time. No expression of pluripotency markers was observed. Early = 1–3 in vitro; mid = days 6–7 in vitro; late = days 14–16 in vitro. Nuclei stained with Hoechst (blue). Scale bar = 100 μm. (PDF 562 kb) [file 13287_2019_1255_MOESM5_ESM.pdf]

## Supplementary Figure 6

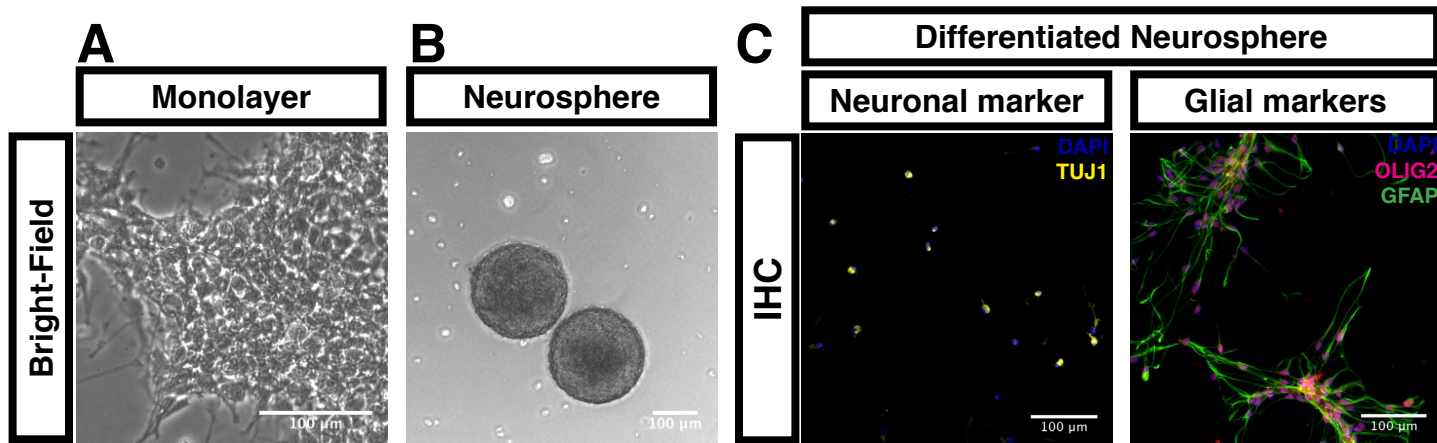

Supplement: Supplementary file 6 — Figure S6. drNPC-derived spheres can be derived from monolayers and give rise to neurons and glia in vitro. Dissociating 80% confluent drNPC monolayers and plating them at 10 cells/μl density results in free-floating spheres within 7 days of culturing. Differentiating spheres for additional 7 days in the presence of serum will result in GFAP-positive astrocytes, Olig2-positive oligodendrocytes, and Tuj1-positive neurons. Scale bar = 100 μm. Representative bright field images of drNPC monolayer (left) and spheres (centre), and a representative IHC image of differentiated drNPC-derived spheres. (PDF 249 kb) [file 13287_2019_1255_MOESM6_ESM.pdf]

Supplementary Figure 7

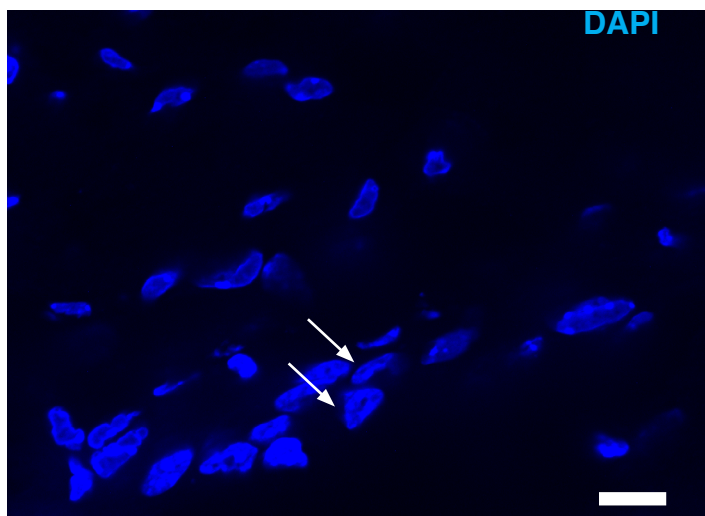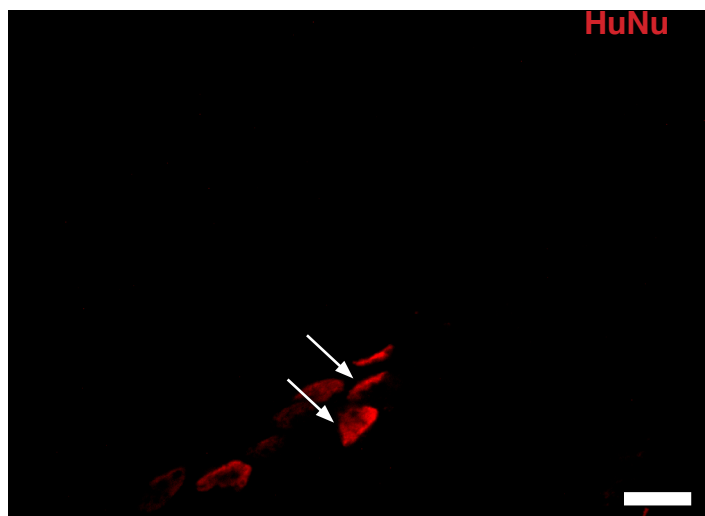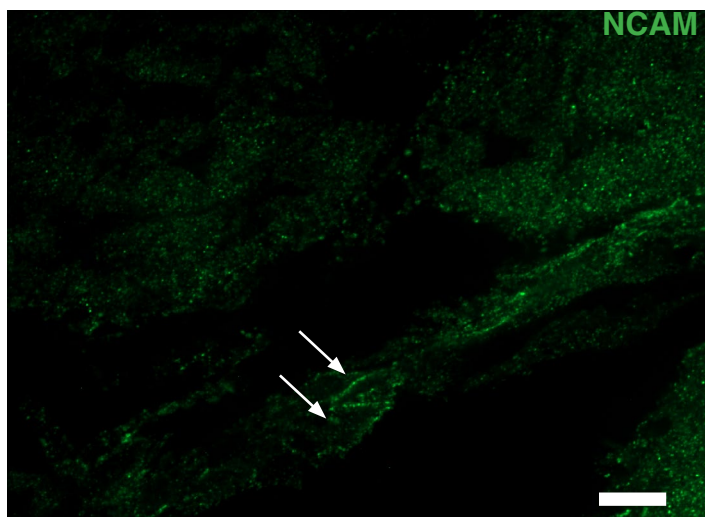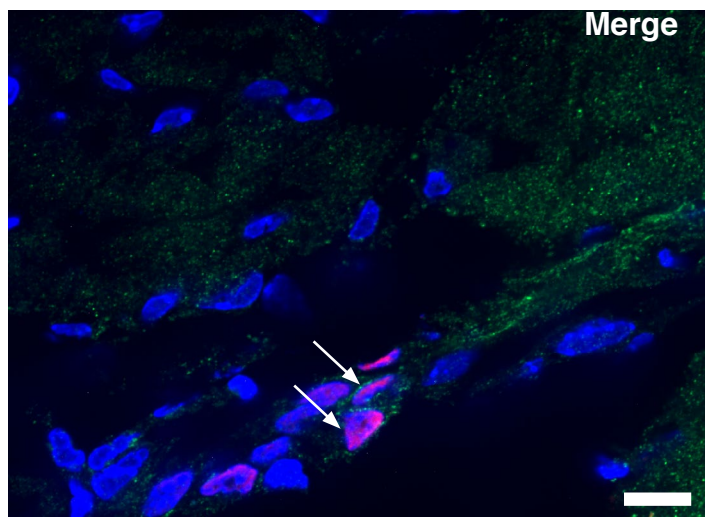

Supplement: Supplementary file 7 — Figure S7. drNPCs present in olfactory bulb express markers of neuronal differentiation. drNPCs transplanted into the SCID/Beige animals are present in the olfactory bulb at 1 month post-transplant and contain a subpopulation of NCAM-positive cells (white arrows). Scale bars = 10 μm. (PDF 831 kb) [file 13287_2019_1255_MOESM7_ESM.pdf]
