# Supplementary material for: Examining the fundamental biology of a novel population of directly reprogrammed human neural precursor cells
Source: Stem Cell Res Ther. 2019 Jun 13;10:166. doi: 10.1186/s13287-019-1255-4 (PMC6567617; doi:10.1186/s13287-019-1255-4)
Supplement: Supplementary file 8 — Figure S8. Pixel colocalization analysis of cytoplasmic immunofluorescent staining. A: Co-localization measurement of cytoplasmic immunofluorescent staining in mouse brain cryosections. Zeiss Zen software was used to measure channel intensities of each immunofluorescently stained pixel and quantify a double positive pixel ratio in defined regions of interest. Threshold pixel intensities were adjusted first to non-labeled areas (Box 1), followed by fluorophore labeled secondary antibody backgrounds (Box 2) in the same section. Pixels of human cytoplasmic epitope-specific STEM121-labeled area (Box 3) was compared to STEM121-negative areas. The sample image, scatter plot of all three regions (Boxes 1, 2, and 3), and pixel measurements/co-localization coefficient are shown. (PDF 371 kb) [file 13287_2019_1255_MOESM8_ESM.pdf]

Supplementary Figure 8

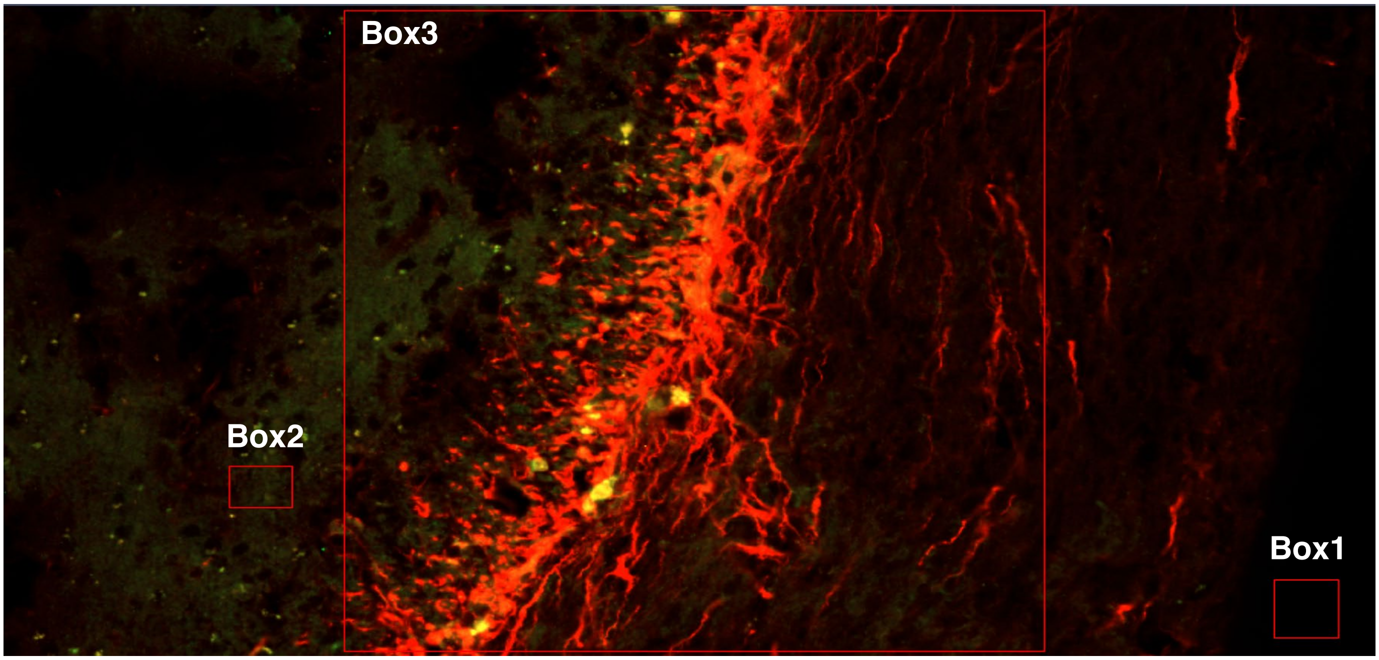

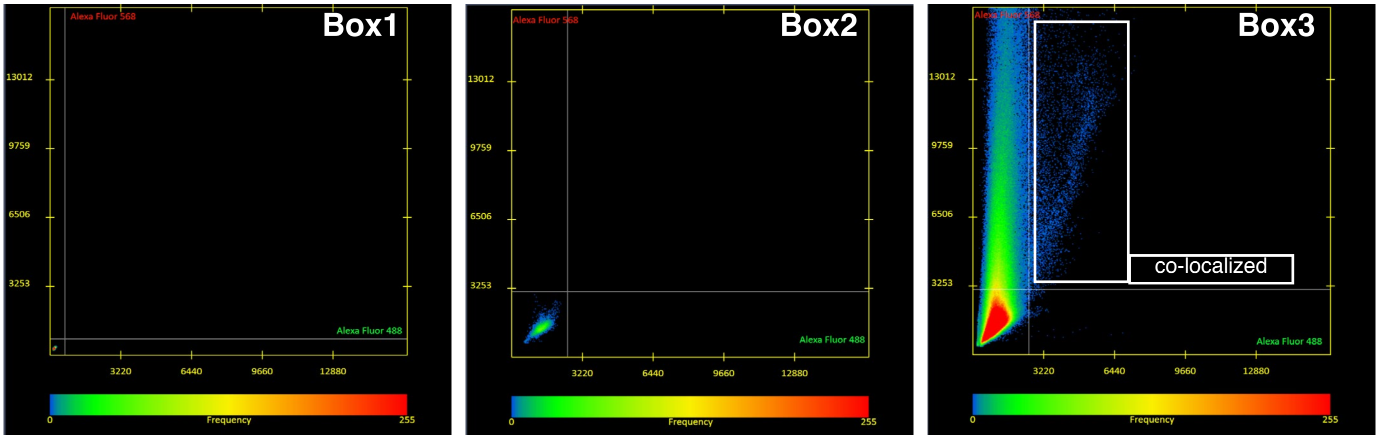

Region	Scatter...	Pixel Count	Area(μm²)	: Coloc Coeff...
A	B	C	D	I
EntireImage	Alexa Fluor 488	573	29.52612	--
EntireImage	Alexa Fluor 568	213110	10981.34519	--
EntireImage	Colocalization	9338	481.1778	0.04198
EntireImage	Background	1850579	95358.48529	--
EntireImage	Global	2073600	106850.5344	1
1 Rectangle	Alexa Fluor 488	0	0	--
1 Rectangle	Alexa Fluor 568	0	0	--
1 Rectangle	Colocalization	0	0	0
1 Rectangle	Background	8827	454.84648	--
1 Rectangle	Global	8827	454.84648	1
2 Rectangle	Alexa Fluor 488	0	0	--
2 Rectangle	Alexa Fluor 568	0	0	--
2 Rectangle	Colocalization	0	0	0
2 Rectangle	Background	6230	321.02567	--
2 Rectangle	Global	6230	321.02567	1
3 Rectangle	Alexa Fluor 488	214	11.02721	--
3 Rectangle	Alexa Fluor 568	204998	10563.34194	--
3 Rectangle	Colocalization	8553	440.72754	0.04005
3 Rectangle	Background	828870	42710.84223	--
3 Rectangle	Global	1042635	53725.93892	1

Co-localization Coefficient

→ 0

→ 0

→ 0.04005
